# Detection of Aortic Valve Opening and Estimation of Pre-Ejection Period in Forcecardiography Recordings

**DOI:** 10.3390/bioengineering9030089

**Published:** 2022-02-22

**Authors:** Jessica Centracchio, Emilio Andreozzi, Daniele Esposito, Gaetano Dario Gargiulo, Paolo Bifulco

**Affiliations:** 1 Department of Electrical Engineering and Information Technologies, University of Naples Federico II, Via Claudio, 21 80125 Napoli, Italy; jessica.centracchio@unina.it (J.C.); daniele.esposito@unina.it (D.E.); paolo.bifulco@unina.it (P.B.); 2 School of Engineering, Design and Built Environment, Western Sydney University, Penrith 2751, Australia; g.gargiulo@westernsydney.edu.au

**Keywords:** forcecardiography, seismocardiography, mechanocardiography, pre-ejection period, systolic time intervals, cardiac monitoring, cardiac function

## Abstract

Forcecardiography (FCG) is a novel technique that measures the local forces induced on the chest wall by the mechanical activity of the heart. Specific piezoresistive or piezoelectric force sensors are placed on subjects’ thorax to measure these very small forces. The FCG signal can be divided into three components: low-frequency FCG, high-frequency FCG (HF-FCG) and heart sound FCG. HF-FCG has been shown to share a high similarity with the Seismocardiogram (SCG), which is commonly acquired via small accelerometers and is mainly used to locate specific fiducial markers corresponding to essential events of the cardiac cycle (e.g., heart valves opening and closure, peaks of blood flow). However, HF-FCG has not yet been demonstrated to provide the timings of these markers with reasonable accuracy. This study addresses the detection of the aortic valve opening (AO) marker in FCG signals. To this aim, simultaneous recordings from FCG and SCG sensors were acquired, together with Electrocardiogram (ECG) recordings, from a few healthy subjects at rest, both during quiet breathing and apnea. The AO markers were located in both SCG and FCG signals to obtain pre-ejection periods (PEP) estimates, which were compared via statistical analyses. The PEPs estimated from FCG and SCG showed a strong linear relationship (r > 0.95) with a practically unit slope, and 95% of their differences were found to be distributed within ± 4.6 ms around small biases of approximately 1 ms, corresponding to percentage differences lower than 5% of the mean measured PEP. These preliminary results suggest that FCG can provide accurate AO timings and PEP estimates.

## 1. Introduction

In recent decades, continuous, non-invasive, mechanical assessment of cardiovascular function has been pursued through alternative techniques to ultrasound, such as Seismocardiography (SCG) [1,2,3], Ballistocardiography (BCG) [4,5,6], Gyrocardiography (GCG) [7,8] and Kinocardiography (KCG) [9,10]. SCG is undoubtedly the most mature of these techniques that is also suitable for wearable applications, and, in fact, it is currently regarded as a reference for cardiomechanical monitoring. SCG is usually performed via small and lightweight accelerometers based on Microelectromechanical System (MEMS) technologies, which are usually placed on different sites of subjects’ chest, such as the xiphoid process, the sternum body, the manubrium, the left and right sternal borders, the left and right clavicles [3]. Most applications that are aimed at performing continuous cardiac monitoring of subjects usually provide for the localization of specific peaks and valleys of the SCG signal, which relate well with various events of the cardiac cycle. Examples of such events are opening and closure of heart valves, isovolumic contraction, cardiac filling and blood injection [2]. The temporal relationship between peaks and valleys of SCG and cardiac cycle events was first thoroughly assessed in the study by Crow et al. [11] via comparison with Echocardiography. This led to the definition of the following nine fiducial markers:

- Peak of atrial systole (AS);
- Mitral valve closure (MC);
- Isovolumic movement (IM);
- Aortic valve opening (AO);
- Isotonic contraction (IC);
- Peak of rapid systolic ejection (RE);
- Aortic valve closure (AC);
- Mitral valve opening (MO);
- Peak of rapid systolic filling (RF);

which are largely recognized as the fundamental SCG markers [2,3]. More recently, further echocardiographic studies have been conducted to characterize the relationship between SCG fiducial markers and cardiac cycle events [12,13,14,15]. In particular, Lin et al. analyzed the SCG signals acquired from the four heart sounds auscultation sites and their relationship with different events of the cardiac cycle, also defining new SCG fiducial markers, e.g., left ventricular lateral wall and septal wall contraction peak velocities and trans-aortic and trans-pulmonary peak flows [12]. The timings of the SCG markers allow estimating time intervals that give important insights into cardiac mechanics, such as pre-ejection period (PEP), left ventricular ejection time, rapid diastolic filling time, isovolumic contraction and relaxation times [11,12,13,14,15,16]. In particular, the PEP, which is commonly defined as the time interval between the onset of the QRS complex (i.e., the Q-wave) in the Electrocardiogram (ECG) signal and the subsequent AO event in the SCG, has been the subject of numerous studies because of its key role in determining the health status of patients with heart failure [17,18,19,20].

In addition to the latest techniques that rely on inertial sensors (e.g., GCG and KCG), a novel technique based on force sensors has recently been proposed: Forcecardiography (FCG). FCG measures the local forces induced on the chest wall by the mechanical activity of the heart and lungs [21,22,23]. The first forcecardiographic recordings were acquired via a custom-designed, piezoresistive force sensor, which had already been used in muscle contraction monitoring [24] and hand gestures recognition [25]. The sensor consists of a Force Sensitive Resistor (FSR), which transduces changes in pressure exerted on its active area into changes in its electrical resistance, equipped with a dome-shaped mechanical coupler, which ensures a good transduction of the forces originating from human tissues to the active area of the sensor. The changes in electrical resistance are finally transduced into voltage signals via a transimpedance amplifier circuit [21]. Simultaneous recordings have been performed via an FSR-based sensor and a MEMS accelerometer, rigidly attached to each other, to compare FCG and SCG signals acquired from the xiphoid process on subjects under resting apnea conditions. The results showed that, in addition to a component (referred to as HF-FCG), which turned out to be very similar to SCG, the FCG signals showed up with a further component (referred to as LF-FCG) that featured large, low-frequency, negative force peaks occurring approximately at the end of the ECG T-waves, which corresponded to forces directed inward. The slow oscillations observed in the LF-FCG signals appear to be associated with ventricular emptying and filling events and cannot be appreciated in SCG, which, therefore, seems not able to provide information about this aspect of cardiac mechanics. The FSR-based FCG sensor has also been tested on subjects at rest while breathing at various rates and has been shown to be suitable for simultaneous cardiorespiratory monitoring [22]. Indeed, the raw signal provided by the FCG sensor consists of two main components: the Forcerespirogram (FRG), which reflects the respiratory activity, and the actual Forcecardiogram, which captures the cardiac activity. In a very recent study, a novel piezoelectric FCG sensor has been proposed for multimodal cardiorespiratory sensing [23]. The sensor can monitor respiration, infrasonic cardiac vibrations and heart sounds, allowing multiple physiological signals to be captured simultaneously from a single point of contact on the chest. In particular, the HF-FCG and HS-FCG (heart sounds component of FCG) signals provided by the piezoelectric FCG sensor turned out to be very similar to SCG and Phonocardiographic (PCG) signals, respectively. Therefore, FCG could be used for the measurement of cardiac time intervals (by detecting the fundamental SCG markers) and, at the same time, of respiration intervals and heart sounds, which cannot be captured by common SCG accelerometers. For this reason, FCG potentially has a much wider spectrum of clinical applications than SCG. However, FCG has not yet been demonstrated to provide the timings of well-established SCG markers with reasonable accuracy, and this currently limits the possibility to obtain the same valuable information provided by SCG.

This study addresses the detection of the AO marker in FCG signals. To this aim, FCG, SCG and ECG signals were acquired simultaneously from three subjects at rest, both during quiet breathing and apnea. The AO markers were located in both SCG and FCG signals to obtain PEP estimates, which were compared via statistical analyses. The results of this preliminary study suggest that FCG is able to provide timings of AO events and estimates of PEP with high accuracy and precision as compared to SCG.

## 2. Materials and Methods

### 2.1. Measurement Setup and Protocol

**Figure 1:**
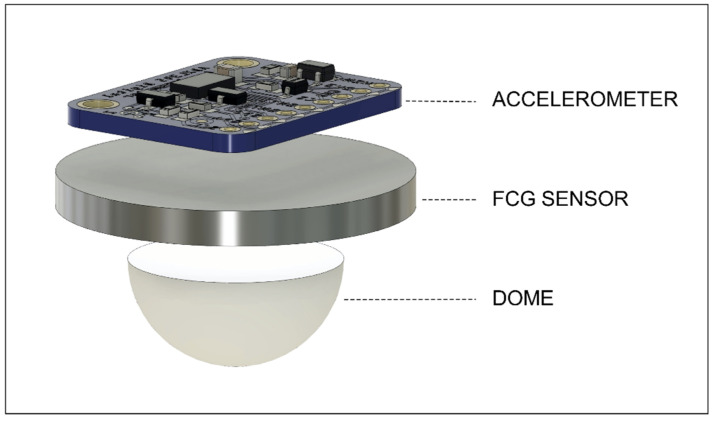
Sensors assembly: piezoelectric FCG sensor with a dome and an MMA7361 accelerometer.

The piezoelectric FCG sensor presented in [23] and a Freescale MMA7361 accelerometer were rigidly fixed to each other to simultaneously acquire FCG and SCG signals from the same point on the chest (see Figure 1). As in [21,22,23], the FCG sensor was equipped with a dome-shaped mechanical coupler that ensures good mechanical transduction from subjects’ skin. A WelchAllyn Propaq^®^ Encore monitor (Welch Allyn Inc., New York, NY, USA) was used to acquire electrocardiographic recordings.

**Figure 2:**
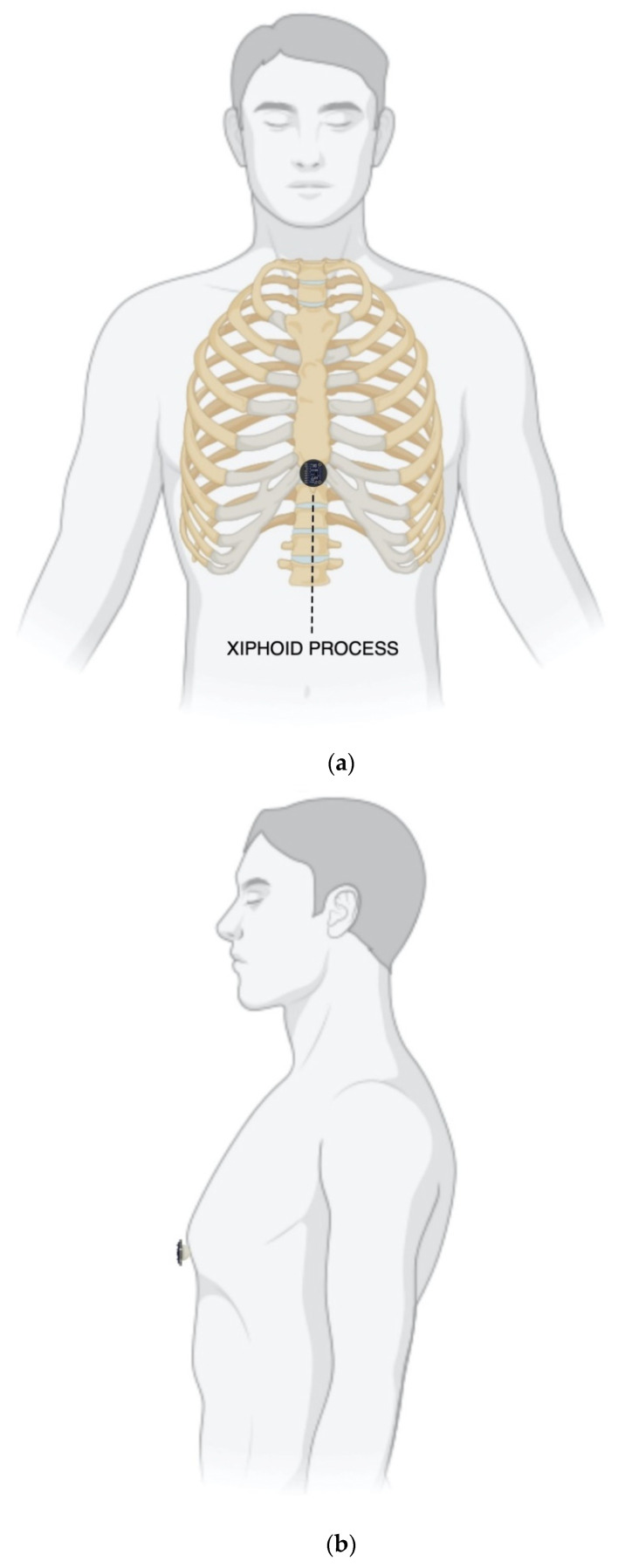
Sensors assembly placement on a subject: (**a**) frontal view; (**b**) lateral view.

Three healthy volunteers (2 males, 1 female, age 29.7 ± 2.52), who signed the informed consent, were asked to comfortably sit on a chair, leaning against the seatback while keeping their back straight. The FCG and SCG sensors assembly was placed onto the xiphoid process of each subject via a medical adhesive tape and then fastened with a belt around the thorax. Figure 2 shows frontal and lateral views of a subject equipped with the FCG/SCG sensors assembly. Simultaneous acquisitions of FCG and dorso-ventral SCG signals, together with an ECG lead I, were carried out via a National Instrument NI-USB4431 DAQ board (National Instruments Corp., 11,500 N Mopac Expwy, Austin, TX 78759-3504, USA), with 24-bit precision and 10 kHz sampling frequency. Multiple acquisitions were performed for each subject, both during quiet breathing and apnea.

### 2.2. Signal Processing

The Forcerespirogram was first subtracted from the raw FCG sensor signals acquired during quiet breathing in order to isolate the actual FCG signal. As in [22,23], the FRG was extracted via a 3rd order Savitzki–Golay filter [26], with a frame length corresponding to about a 1.5 s interval. The actual FCG signals resulting from the respiration signal removal were then band-pass filtered in the 7–30 Hz frequency band via a 2nd order zero-lag Butterworth filter to extract the HF-FCG component. The raw FCG sensor signals acquired during apnea were directly band-pass filtered to obtain the HF-FCG components. The first derivatives of the HF-FCG signals thus obtained were finally computed and referred to as dHF-FCG (the derivatives were computed as finite forward differences). The dorso-ventral SCG signals were obtained from the raw *z*-axis acceleration signals via the same 2nd order zero-lag Butterworth filter used to extract the HF-FCG components from the FCG signals.

### 2.3. Morphological Comparison between FCG and SCG Signals

The ECG-triggered ensemble averages (synchronized with R-peaks) of SCG, HF-FCG, dHF-FCG and ECG signals acquired in apnea conditions were computed. To this aim, the R-peaks were first located in the ECG signal via the “BioSigKit” MATLAB^®^ toolbox, which implements the well-known Pan and Thompkins algorithm [27]. Then, the normalized cross-correlation indices and the time lags of the ensemble averages of HF-FCG vs. SCG and dHF-FCG vs. SCG were evaluated for each subject. As shown in the Section 3, the dHF-FCG and SCG signals scored both a higher normalized cross-correlation index and a lower time lag; therefore, the dHF-FCG signals were actually used for the estimation of PEP from FCG recordings.

### 2.4. Statistical Analyses on Pre-Ejection Period Estimates

The PEPs were estimated as the intervals between the ECG Q-waves, provided by “BioSigKit”, and the related AO markers located on both SCG and dHF-FCG signals. The AO markers were located by taking advantage of the *a priori* knowledge of the R-peaks locations [23], and the missed AO events were annotated for each subject. Regression, correlation and Bland–Altman analyses of the PEP estimates obtained from SCG and dHF-FCG signals were carried out via the MATLAB^®^ function “bland-altman-and-correlation-plot” [28].

## 3. Results

### 3.1. Morphological Comparison between FCG and SCG Signals

**Figure 3:**
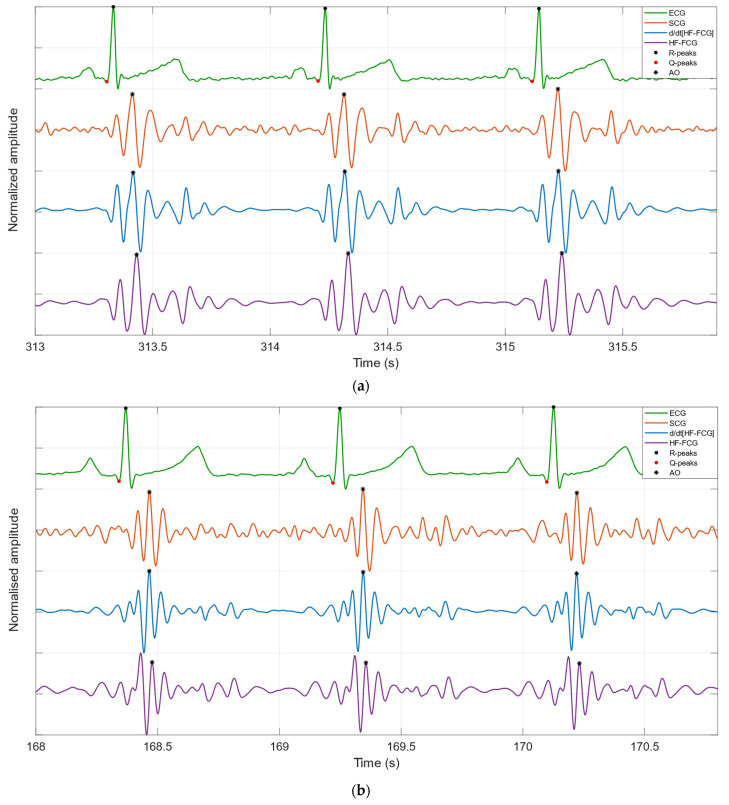
Examples of HF-FCG, dHF-FCG, SCG and ECG signals from (**a**) subject #1 and (**b**) subject #3.

Some excerpts of HF-FCG, dHF-FCG, SCG and ECG signals from subjects #1 and #3 are depicted in Figure 3. It could be noted by visual inspection that the HF-FCG is lagged with respect to the SCG, and their peaks and valleys do not match very well. Indeed, the peaks and valleys of SCG appear as corresponding to the points of maximum slope of the HF-FCG. The dHF-FCG, on the other hand, is practically synchronous with the SCG and features peaks and valleys that match those of SCG remarkably well.

**Figure 4:**
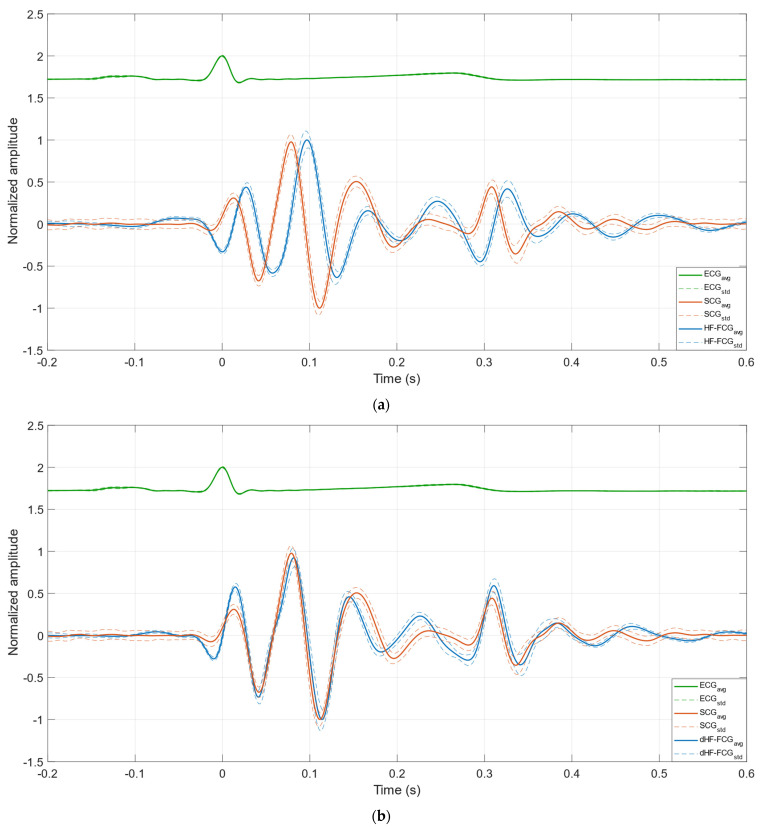

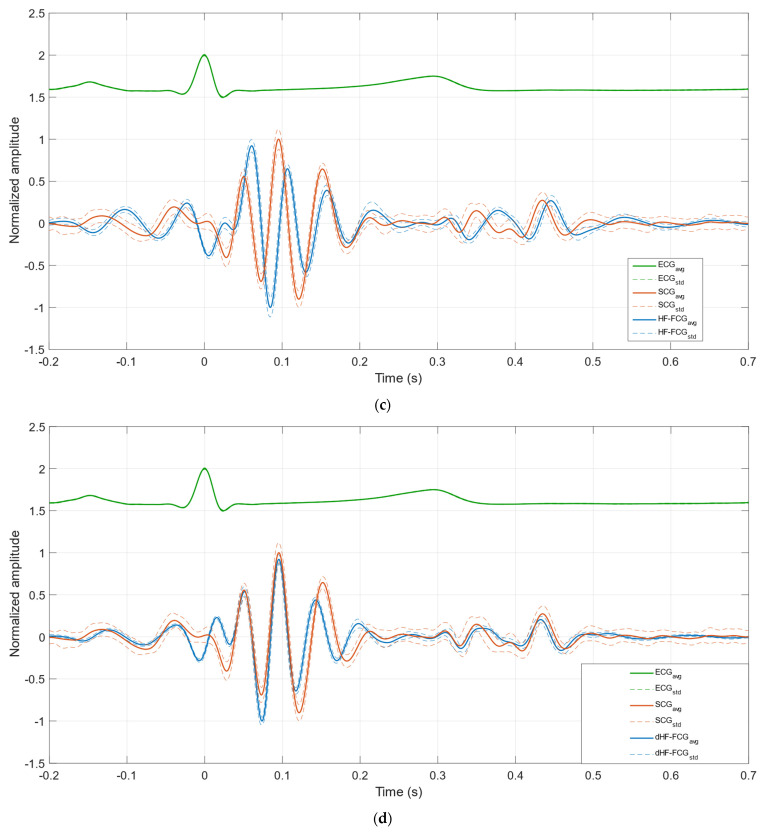
(**a**) Ensemble averages of HF-FCG, SCG and ECG of subject #1; (**b**) ensemble averages of dHF-FCG, SCG and ECG of subject #1; (**c**) ensemble averages of HF-FCG, SCG and ECG of subject #3; (**d**) ensemble averages of dHF-FCG, SCG and ECG of subject #3. The ensemble averages are depicted as solid lines, while the limits of the ± SD ranges are depicted as dashed lines.

Figure 4a,b depict, respectively, the ensemble averages of HF-FCG, SCG and ECG and of dHF-FCG, SCG and ECG for subject #1, while Figure 4c,d depict the same signals for subject #3. Table 1 reports the normalized cross-correlation indices and the time lags for each subject. As a reconfirmation of what has been observed in Figure 3, in Figure 4, it can be noticed that the ensemble averages of dHF-FCG and SCG showed up with almost the same peaks and valleys. Moreover, dHF-FCG and SCG scored, at the same time, higher normalized cross-correlation indices and lower time lags with respect to HF-FCG and SCG (on average, 0.80 vs. 0.87, 14.7 ms vs. −0.7 ms). These results, which are consistent across the subjects involved in the study, suggest that the first derivative of the HF-FCG signal captures the salient features of the SCG signal better than the HF-FCG signal itself. For this reason, the dHF-FCG was used to locate the AO events and obtain the PEP estimates to be compared with the SCG ones.

### 3.2. Statistical Analyses on Pre-Ejection Period Estimates

In Table 2, the number of heartbeats detected in ECG, as well as the number of missed AO events detected in SCG and dHF-FCG, both in quiet breathing and apnea tests, are reported for each subject. According to these results, the SCG and dHF-FCG scored a sensitivity of 100% and 99.8% in apnea and quiet breathing conditions, respectively.

**Figure 5:**
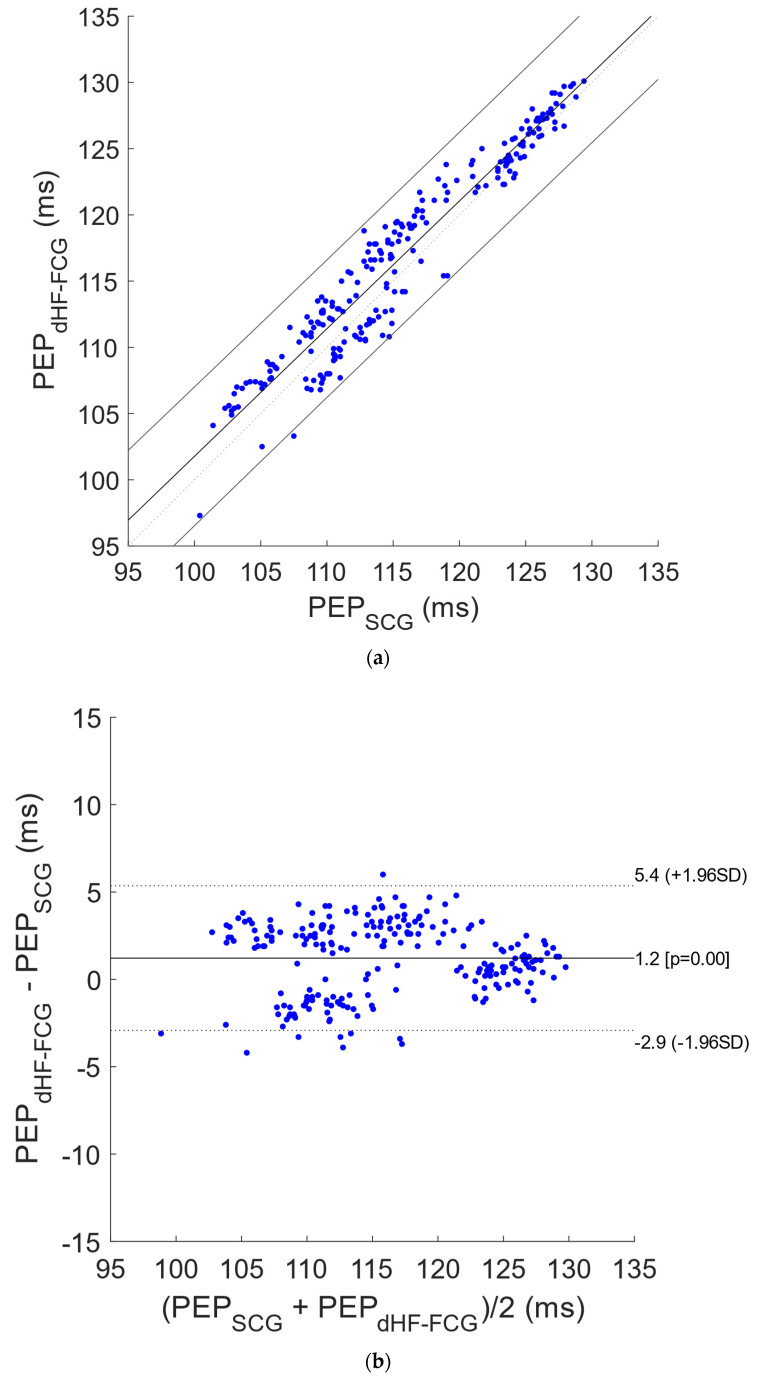
Statistical analyses on PEP estimates related to signals acquired during apneas: (**a**) results of regression and correlation analyses; (**b**) results of Bland–Altman analysis.

**Figure 6:**
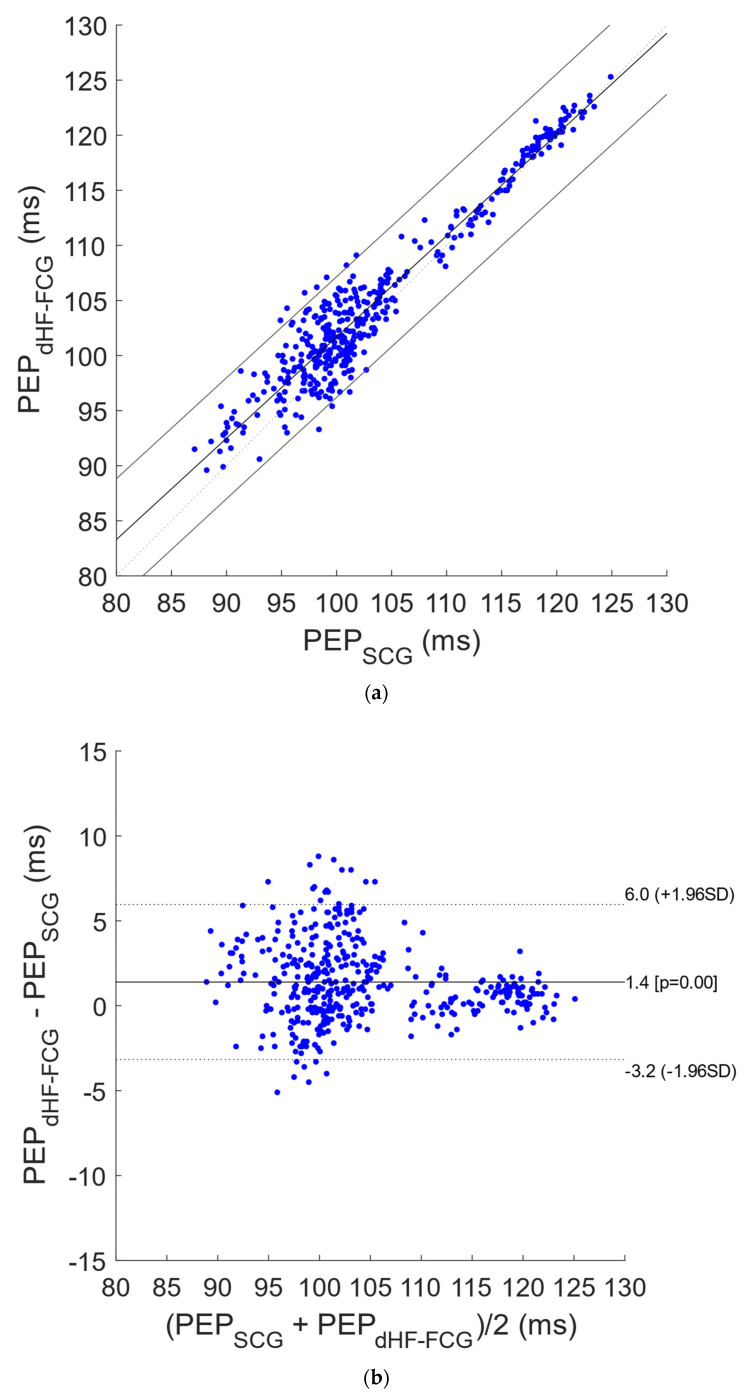
Statistical analyses on PEP estimates related to signals acquired during quiet breathing: (**a**) results of regression and correlation analyses; (**b**) results of Bland–Altman analysis.

Figure 5 shows the results of the regression, correlation and Bland–Altman analyses that were performed on a total of 227 PEP estimates obtained from SCG and dHF-FCG signals acquired during apnea tests. The statistical analyses reported a slope and intercept of 0.964 and 5.4 ms (R^2^ = 0.92) and a bias of 1.2 ms (*p* < 0.0001) with limits of agreement of (−2.9; 5.4) ms. In Figure 6, the results of statistical analyses of PEP estimates obtained from signals acquired during quiet breathing are depicted. The analyses were performed on a total of 423 PEP estimates and reported a slope and intercept of 0.919 and 9.8 ms (R^2^ = 0.92), as well as a bias of 1.4 ms (*p* < 0.0001), with limits of agreement of (−3.2; 6.0) ms.

## 4. Discussion

This study focused on the detection of the aortic valve opening events in FCG signals, which were acquired via the piezoelectric FCG sensor presented in [23], together with SCG and ECG signals. The HF-FCG component was extracted from the FCG signals via zero-lag band-pass filtering, which was used to process also the SCG signals. The morphologies of the HF-FCG signal and of its first derivative (dHF-FCG) were compared with the morphology of the SCG signal by evaluating the normalized cross-correlation index and the time lag of their ECG-triggered ensemble averages (synchronized with the R-peaks). The results of this morphological analysis showed that dHF-FCG exhibited the highest similarity with SCG. Indeed, from a quantitative point of view, dHF-FCG scored both higher normalized cross-correlation indices and lower time lags with SCG; from a qualitative point of view, the dHF-FCG signals showed up with peaks and valleys almost matching those of the SCG signals. Moreover, from visual inspection of Figure 3 and Figure 4, it could be noticed how peaks and valleys of SCG corresponded to points of maximum positive/negative slope of HF-FCG. This finding suggests the existence of a derivative relationship between SCG and HF-FCG, which could explain the higher time lags observed between them, as well as the higher similarity between SCG and the first derivative of HF-FCG. The reason behind this derivative relationship is currently not clear and may lie in the dynamic response of the piezoelectric sensor; this phenomenon undoubtedly deserves an in-depth investigation, which is out of the scope of this research and will be addressed in future studies.

Based on these results, the dHF-FCG was actually used to locate the AO events in the FCG recordings and then to obtain the PEP estimates to be compared with those extracted from SCG. The measurements of PEP obtained from FCG and SCG showed a strong linear relationship (r > 0.95) with a practically unit slope, and 95% of their differences were found to be distributed within ±4.6 ms around small biases of approximately 1 ms, corresponding to percentage differences lower than 5% of the mean measured PEP. These preliminary results, as compared to the results of other studies that analyzed the performances of different techniques for PEP estimation [14,29,30], suggest that FCG can provide accurate measurements of PEP in subjects at rest. Indeed, Dehkordi et al. [14] reported that 95% of the differences between PEP estimates obtained via SCG and Echocardiography were distributed within ±25 ms around biases of about 2 ms, and, based on these results, they concluded that SCG provides acceptable accuracy and precision in estimating cardiac timings. Su et al. [29] found similar results concerning the 95% interval of the differences between PEP measurements obtained from an ankle-brachial device as compared to Echocardiography, although with a significantly higher bias of about 30 ms and correlations lower than 0.7, and they concluded that the device under test turned out to be a good alternative to Echocardiography in the evaluation of left ventricular systolic dysfunctions based on the estimation of PEP and other systolic time intervals.

This study has some limitations. The data analyzed were acquired only on three healthy subjects at rest. Therefore, the results should be considered as preliminary and need to be confirmed on a larger cohort of subjects, including subjects performing different activities (e.g., walking, speaking, doing sports). Furthermore, FCG was compared with SCG due to its wide use for PEP monitoring in wearable applications, but SCG is not yet regarded as a gold standard for PEP estimation, so only the agreement between the two techniques could be evaluated. Hence, a comparison of FCG with echocardiographic measurement is envisioned in future studies to assess the performances of FCG in AO detection and PEP estimation against an actual gold standard.

## Figures and Tables

**Table 1:** Normalized cross-correlation indices (NCC) and time lags between the ensemble averages of HF-FCG vs. SCG and dHF-FCG vs. SCG for each subject. Positive time lags corresponded to FCG signals delayed with respect to SCG.

Subject	HF-FCG vs. SCG		dHF-FCG vs. SCG	
	NCC	Lag (ms)	NCC	Lag (ms)
#1	0.8333	18.6	0.9107	1.2
#2	0.7764	14.7	0.8988	−2.2
#3	0.7773	10.7	0.7998	−1.2

**Table 2:** Number of heartbeats in ECG and of missed AO events in SCG and in dHF-FCG for each subject in quiet breathing and apnea conditions.

Subject	Heartbeats in ECG		Missed AO in SCG		Missed AO in dHF-FCG	
	Apnea	Quiet Breathing	Apnea	Quiet Breathing	Apnea	Quiet Breathing
#1	112	200	0	0	0	0
#2	54	118	0	0	0	0
#3	61	106	0	1	0	1

## Data Availability

The datasets presented in this article are not readily available because informed consent from the subjects involved was obtained only for this study and not for public availability. Requests to access the datasets should be directed to E.A. (emilio.andreozzi@unina.it) or P.B. (paolo.bifulco@unina.it).

## Abbreviation

List of abbreviations and their definitions.

Abbreviation	Definition
AC	aortic valve closure
AO	aortic valve opening
AS	peak of atrial systole
BCG	ballistocardiography
dHF-FCG	first derivative of HF-FCG
ECG	electrocardiogram
FCG	forcecardiography
FRG	forcerespirogram
GCG	gyrocardiography
HF-FCG	high-frequency component of FCG signal
HS-FCG	heart sounds component of FCG signal
IC	isotonic contraction
IM	isovolumic movement
KCG	kinocardiography
LF-FCG	low-frequency component of FCG signal
MC	mitral valve closure
MEMS	microelectromechanical systems
MO	mitral valve opening
PCG	phonocardiography
PEP	pre-ejection period
RE	peak of rapid systolic ejection
RF	peak of rapid systolic filling
SCG	seismocardiography
